# Applied machine learning as a driver for polymeric biomaterials design

**DOI:** 10.1038/s41467-023-40459-8

**Published:** 2023-08-10

**Authors:** Samantha M. McDonald, Emily K. Augustine, Quinn Lanners, Cynthia Rudin, L. Catherine Brinson, Matthew L. Becker

**Affiliations:** 1 Department of Chemistry, Duke University, Durham, NC USA; 2 Thomas Lord Department of Mechanical Engineering and Materials Science, Duke University, Durham, NC USA; 3 Department of Biostatistics and Bioinformatics, Duke University, Durham, NC USA

**Keywords:** Biomedical materials, Computer science, Biomaterials, Biomedical engineering, Polymer synthesis

## Abstract

Polymers are ubiquitous to almost every aspect of modern society and their use in medical products is similarly pervasive. Despite this, the diversity in commercial polymers used in medicine is stunningly low. Considerable time and resources have been extended over the years towards the development of new polymeric biomaterials which address unmet needs left by the current generation of medical-grade polymers. Machine learning (ML) presents an unprecedented opportunity in this field to bypass the need for trial-and-error synthesis, thus reducing the time and resources invested into new discoveries critical for advancing medical treatments. Current efforts pioneering applied ML in polymer design have employed combinatorial and high throughput experimental design to address data availability concerns. However, the lack of available and standardized characterization of parameters relevant to medicine, including degradation time and biocompatibility, represents a nearly insurmountable obstacle to ML-aided design of biomaterials. Herein, we identify a gap at the intersection of applied ML and biomedical polymer design, highlight current works at this junction more broadly and provide an outlook on challenges and future directions.

## Abstract (web summary)

The design of polymers for regenerative medicine could be accelerated with the help of machine learning. Here the authors note that machine learning has been applied successfully in other areas of polymer chemistry, while highlighting that data limitations must be overcome to enable widespread adoption within polymeric biomaterials.

## Introduction

**Fig. 1:**
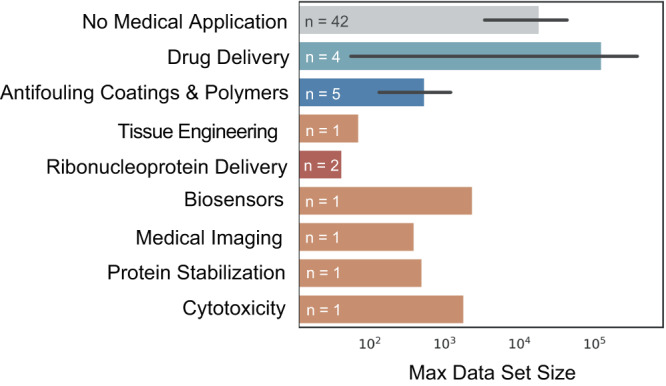
Data set sizes documented for applications within polymer chemistr**y**. There are only a few examples of ML applied to biomedical polymer questions (*n* = the number of papers for each application). Data availability is a considerable concern for ML approaches within many biomedical applications as demonstrated by the relatively few number of papers which have been published on this topic as well as the small size of the data sets used in these existing approaches.

Many of the machine learning (ML) approaches at the intersection of medicine and chemistry focus on small molecule synthesis for drug discovery^1–7^. As shown in Fig. 1, there is a considerable gap in strategies targeting polymers in medicine despite medical polymers representing an 18.4-billion-US dollar global market as of 2021– appearing in diverse applications such as catheters, coatings, implants, etc^8–10^.

Among the bottlenecks experienced in the development of commercial medical polymers, the research and design of such materials represent a huge investment of time, money, and energy. New materials are often designed through experimental intuition and further developed through trial-and-error synthesis. This process is not only economically inefficient, but also often fails to produce polymers that have all the target properties for an intended application. This is due, in part, to the fact that polymers exhibit less predictable/intuitive structure-property relationships than small molecule counterparts due to the greater number of variables which dictate their properties—namely, composition, molecular mass, intermolecular forces, and architecture^11,12^. Even within a class of compositionally similar polymers, these relationships can be nonlinear and hard to identify, which makes it difficult to design materials for a specific property outcome. Thus, employing ML as a tool that is sensitive to patterns in data which are critically important, but indiscernible to humans, could accelerate the development of translationally relevant polymers^13^.

Most work at the intersection of ML and polymer design more broadly can be separated into two general tasks:

1. *Property prediction (or forward problem design):* Given a polymer structure, predict specified properties of interest. This approach is highly valuable for the screening of candidate materials, enabling synthesis of the most promising candidate rather than the whole library.
2. *Structure generation (or inverse problem design):* Given properties of interest, predict polymeric structures that may demonstrate the desired properties. This strategy can expedite the materials design process and remove limitations set by user intuition.

Applications of ML are not limited to polymer design and have also been successfully implemented for process optimization and advancing our understanding of structure-property relationships within these complex systems. Ultimately, accessing the next generation of polymeric biomaterials will more than likely require some combination of these approaches. To quote Anne Fischer, a DARPA program manager for accelerated molecular discovery:

*“It’s not about replacing chemists. It’s about giving chemists the tools to allow them to implement and apply the chemistry and allow them to be creative high-level thinkers.”* ^14^

### Challenges with data availability

Advancements in property prediction and inverse modeling in related fields have been driven primarily by relatively data-hungry supervised learning algorithms, including deep learning, random forests, gaussian process models^15–18^. Such models are very promising for developing advancements in polymer design given the complexity of polymer systems. However, they often require more labeled data than the typical experimentalist produces to discover more fine-tuned interactions with limited prior understanding of the system.

As such, data availability represents a primary obstacle for this field irrespective of the approach^13^. Experimental datasets are often small (on the order of 1–20 unique structures) and incompatible with each other due to differences in experimental methods and/or data analysis. Property handbooks which serve as reliable references for the experimentalist are often compiled with only the polymer name or with limited structural information^19,20^. Polymers are not always named according to the conventions of the International Union of Pure and Applied Chemistry, which makes the name a poor representation of the materials’ structure. Additionally, these sources typically are not easily exportable, which is necessary for implementation in code. Online databases, as shown in Table 1, contain thousands of polymers with some of their associated properties, structural images, and some additional methods of identification^13,21–23^. However, both polymer handbooks and online databases suffer from high data sparsity and are limited in the properties they contain. This limits the properties that can be predicted with supervised learning. For example, it would be difficult to create a labeled dataset for training a model to predict degradation time given that this property is often not included in current databases. This also affects what properties can be used as input features (e.g., molecular mass) which may limit prediction performance. Additionally, these data sources are often not accessible—frequently requiring an academic affiliation, have no easy way to download the data, and/or expect a fee before use.

**Table 1:** Summary of current online databases

Database^Ref^	# of Polymers	Polymer Class(es)	Properties	Drawbacks
PolyInfo^113^^a^	31,495	Diverse	Physical, optical, thermal, electrical, physicochemical, rheological, solution, mechanical	
Khazana^23^^b^	965	Conjugated polymers, commercial thermoplastics, polyesters	Electrical, optical	
Polymers: a Property Database^114^^a^	30,000	Diverse	Physical, optical, thermal, electrical, physicochemical, rheological, mechanical	
Polymer Property Predictor and Database^115^^a^	212–6524	Conjugated polymers, commercial thermoplastics	Solution, thermal	
MatWeb^116^^a^	97,635	Commercially available polymers	Physical, optical, thermal, electrical, physicochemical, rheological, mechanical	
Block Copolymer Phase Behavior Database^117^^a^	5300	Block copolymers	Phase measurements for block copolymers	-
Electron Affinity and Ionization Potential Data^62^^c^	42,966	Halogenated polymers, conjugated polymers, charged polymers	Copolymer electron affinities and ionization potentials	-

= fee or academic affiliation required,  = no public API or ability to download,  = no structure information,  = unstandardized/text entries,  = cannot download the whole dataset/can only download a subset.

^a^experimental data.

^b^experimental and simulated data.

^c^simulated data.

Experimental datasets containing properties of interest (e.g., in vivo degradation time, cytotoxicity) for the design of biomedical polymers specifically demonstrate high scarcity with respect to the number and size of available datasets. Figure 1 shows efforts in ML applied to biomedical polymers by application. This scarcity persists for many reasons, including the time and cost associated with characterizing in vivo properties, lack of standardization within in vitro methods, and the lack of applicability of some biomedical properties in other fields of interest to polymers (e.g., drug release profile has little applicability for energy or sustainable materials).

Previous work in ML applied to polymer design has addressed data availability concerns by simulating data^24–26^. This strategy is particularly promising for generating labeled datasets for properties which are not commonly characterized because they are difficult to characterize or only relevant for niche applications. Batra et al. used simulated data to train a property prediction algorithm as part of an inverse design approach. In particular, they used density functional theory composition to simulate polymer bandgap labels, one of the target properties in their approach, since experimental values of this property are less common in the literature^25^. Existing molecular dynamics simulations of biomedical properties, such as cytotoxicity, could be used to generate datasets for supervised ML and demonstrate the utility of simulating other medical properties^27,28^. However, it should be noted that this approach can result in error propagation through to the final algorithm, so special care should be taken to confirm the fidelity of the simulation results.

Transfer learning builds a ML model by using a smaller dataset to finetune or adapt a model that was originally trained for a similar task on a larger dataset^29^. This approach has been extremely successful in other fields and within chemistry for overcoming data limitations and improving model performance^29–32^. Using transfer learning, a model pretrained on a large simulated dataset can be finetuned using a smaller experimental dataset to address error propagation concerns, while still reducing the amount of necessary experimental data. Examples of transfer learning for scarce properties from models trained on physically related properties also present a route to developing stronger models with less experimental data^33^. However, transfer learning still often requires more data than the typical experimentalist generates.

Other approaches have leveraged high throughput or automated experiments to generate data more quickly than traditional synthesis^34–36^. The most common high-throughput strategies use continuous-flow systems, plate-based methods, or reactor arrays to run many polymerization reactions simultaneously^37–44^. However, other experimental setups, such as microfluidic reactors or PCR thermocycler setups, may be favorable depending on the property of interest and the number of variables which must be controlled^45,46^. These principles can be automated via programmable robots to further eliminate the need for human intervention and to run reactions at times unfavorable for researchers^43,44,47^. High-throughput approaches are generally promising for properties which can be measured directly with small quantities of polymer (e.g., water uptake, cytotoxicity) and can be combined with methods which can accommodate relatively small datasets, such as active learning or Bayesian optimization^36,48,49^. Within regenerative medicine, high-throughput methods have shown promise for the efficient generation of polymer excipients, and antimicrobial polymer discovery^40–42^. Generating datasets via these existing approaches could be a more immediately achievable way to apply ML to biomedical polymer design. However, these strategies may be hard to scale for properties which require larger quantities of polymer and/or which require processing steps, but can still be employed to reduce the active synthesis time for experimentalists.

Additionally, chain-growth polymerizations, such as ring-opening polymerization and reversible addition fragmentation chain transfer polymerization, have been the methods of choice for high-throughput experiments^37–46^. There is a notable gap of high-throughput strategies for step-growth polymerizations even though these structures are common among commercially available medical polymers and are of interest for developing new medical polymers due to backbone heteroatoms which enable degradability. Poly(lactic acid) (PLA), poly(urethanes) and nylons are classes of polymers commonly synthesized via step-growth polymerization methods and employed in a diverse range of commercial medical applications, including medical tubing, short-term implants, and sutures^50–53^. Despite this, there are only a couple of examples of high-throughput strategies which target step-growth type reactions^47,54^. Thus, the development of scalable high-throughput methods which include step-growth polymerizations and biomedical properties would greatly advance the available data for applied ML.

Ultimately, the need for an accessible, high-quality data source is undeniable. The recently released Community Resource for Innovation in Polymer Technology (CRIPT) is one potentially scalable architecture which shows immense promise toward this goal, focusing on the curation of current and future data rather than trying to solve the curation of historical data^55^. However, the success of this approach is contingent on widespread contribution to the database by experimentalists and the current number of polymers contained in the database is unclear. Additionally, emerging databases must still address polymer naming inconsistencies which has driven the use of BigSMILES strings and knowledge graphs as labels in lieu of traditional names^56^. The adoption of alternative labeling conventions should be widespread to facilitate interoperability between databases and researchers’ familiarity with these new labels. Guidelines for and strong examples of data-sharing are outlined in the FAIR principles. Briefly, these principles identify good data-sharing platforms as being: Findable, Accessible, Interoperable, and Reusable^57^. Updating existing platforms to abide by these principles would considerably lower the barrier to applying ML to a broader set of polymer design problems. Similarly, these principles should be at the core of future data-sharing ventures.

### Encoding chemical information

Encoding the chemical information of polymers into a machine-readable format is an essential and nontrivial step for ML in this area. Polymer structures are complex inputs which have been represented through molecular graphs, monomer simplified molecular-input line-entry system (SMILES), and polymer SMILES representations (BigSMILES, curlySMILES, etc)^13,58,59^. SMILES representations have been a popular choice as they can be one-hot encoded (a group of bits among which the combinations of values are only those with a single high [1] bit and all the others low [0]) and are more easily interpreted by experimentalists who may not have strong computer science skills. Most approaches opt for training algorithms on repeat unit SMILES which makes it difficult to deal with structures which contain more than one repeat unit (e.g., copolymers) or exhibit complex architecture (e.g., star-shape)^33,60^. BigSMILES and curlySMILES represent polymer extensions which factor in the limitations intrinsic to the original SMILES syntax. However, these representations are not all-inclusive and may require passing additional variables, such as monomer stoichiometry and polymer dispersity. Similarly, the encoding of SMILES strings results in a high dimensional input which is not suitable for all ML approaches depending on the complexity of the model and data set size.

Molecular descriptors can be used in lieu of an encoded SMILES input to reduce the number of input features. This approach works well for systems which have low structural diversity and/or with algorithms that cannot accommodate encodings with connectivity information, such as linear models, support vector machines and random forest^61^. As discussed in the *Potentially Helpful Tools* section, existing Python packages have been developed to automate the generation of these descriptors.

Graph representations have also demonstrated promising success as featurization methods which capture other polymer features (e.g., monomer stoichiometry, molar mass distribution) in addition to connectivity information^62^. While these methods are less interpretable than string counterparts, they are more complete representations of polymer systems.

### Potentially helpful tools

An abundance of Python packages for chemistry have been comprehensively curated into a repository on GitHub^63^. Many tasks have been tackled through these packages including, but not limited to structure representation (e.g., RDkit), database wrappers (e.g., pubchempy, ChemSpiPy) and atomistic simulations. Table 2 shows a collection of Python packages focused particularly on polymer chemistry. E3 and E4, may be helpful in generating simulated datasets. Other packages like XenonPy or the BigSMILES parser (Table 2 E1 and E5) can be incorporated into approaches directly to reduce development time.

**Table 2:** Python polymer packages

Entry	Package^Ref^	Description
E1	XenonPy^32^	Various pretrained models for different properties of interest and other ML tools related to chemical problems (including polymers)
E2	Topoly^118^, PSP^119^, PysoftK^120^	Toolkits for polymer topology and structure
E3	pysimm^121^, Polyply^122^, m2p^123^	Open-source polymer simulations and chain generation
E4	PMD^124^	High throughput molecular dynamic simulations for a variety of properties
E5	BigSMILES Parser^125^	Parses BigSMILES strings
E6	Matminer^126–138^	Materials data miner from online databases

### Current work in ML applied to polymer chemistry

Property prediction and inverse design, as discussed in the following section, represent the two most prominent supervised tasks within machine learning applied to polymer chemistry. Unsupervised methods, such as self-organizing maps, are much less common within polymer chemistry but can be used to explore structure-property correlations and visualize high dimensional data^36, 64^. While this section does not discuss implementation at length, Meyer et al. present a thorough tutorial on how to apply ML to questions within polymeric biomaterials and model selection has been discussed more broadly elsewhere^65–67^. While Python is the language of choice for ML applied to chemical questions, other languages, like R and C++, can be used to build ML models. It should also be noted that ML methods are in an era of explosive growth, which may provide creative solutions to problems not yet solved within polymer chemistry.

### Property prediction

**Fig. 2:**
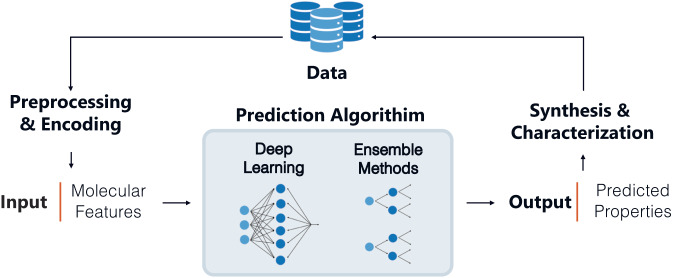
General ML workflow for property prediction tasks. Data (i.e., polymers with known properties) must be preprocessed and encoded before passing desired input (e.g., encoded chemical structure, molecular descriptors) into a prediction algorithm. Irrespective of algorithm choice, training proceeds by tuning the model hyperparameters to minimize prediction error. The trained algorithm can then be used to screen polymer candidates prior to experimental synthesis & characterization. While deep learning and ensemble methods are the most widely used, other supervised methods have been employed (see Table 3) and may be preferred based on the application.

Property prediction tasks (workflow described in Fig. 2) represent the most explored area of this emerging field due to the straightforward problem statement, the accessibility of easy to implement algorithms, and the potential to achieve useful results even with smaller datasets (Table 3). These tasks can be considered on their own as proof-of-concept work, implemented for candidate screening and/or used to develop quantitative structure property relationships. It should be noted that it is important to be careful drawing quantitative structure-property relationships from predictive models because, unless carefully designed, model feature importance alone does not signify causal importance. As shown in Table 3, thermal properties are a popular target in property prediction tasks due to their relevance to a variety of fields, a greater degree of standardization between data sources, and their characterization for most new polymers in the literature. Outside of commonly characterized properties, property prediction tasks are only beginning to be applied to properties of exclusive interest to biomedically relevant polymers, physiological degradation time and biocompatibility (see ML for polymer chemistry in medical applications section).

**Table 3:**
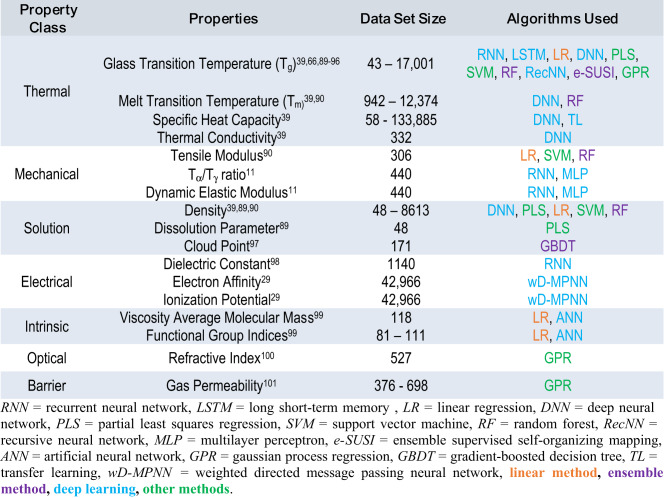
Summary of approaches towards property prediction

Random forest and recurrent neural network (RNN) algorithms have been the primary choice for polymer property prediction tasks (see Table 3). This is symptomatic of the input data and the size of the chosen data set. Random forests and other ensemble methods can discover nonlinear interactions while often not requiring as much data as deep learning algorithms to perform well. Additionally, RNNs perform well with textual input data, which is advantageous for approaches which use a SMILES string to encode polymer structures. In both cases, existing Python packages facilitate the easy implementation of these models as much of the mathematical ‘nitty gritty’ has been abstracted away, allowing users to simply call and apply the algorithms to a dataset of choice. However, ease of implementation should be balanced with an understanding of the limitations associated with the chosen ML method to ensure correct usage.

Investigation into other ML approaches could expand this task with respect to model performance and/or the accessibility of the results. Physics-informed models can improve prediction accuracy and model robustness in smaller data regimes^68,69^. The underlying physics of a system can be introduced through data augmentation. Interventions in the model architecture can also be guided by domain knowledge, and/or introducing constraints (often on the model’s loss function) based on what is physically possible^69^. For example, Bradford et al. included the Arrhenius equation in the final layer of their predictive model to improve the performance of ionic conductivity predictions for polymer electrolytes^68^. These types of improvements to the model architecture would benefit greatly from collaboration between polymer chemists and ML experts.

Compared to black-box approaches (i.e., models which do not provide information about feature importance like deep learning methods), interpretable ML algorithms (i.e., models which information about feature importance can be extracted like random forest algorithms) create models that are more transparent, easier to troubleshoot, and facilitate understanding of the chemical system^70,71^. Alternatively, black-box methods can be supplemented with models like Shapely Additive exPlanations (SHAP) and Locally Interpretable Model-agnostic Explanations (LIME) to assist in the interpretation of predictions^72,73^. These modeling approaches can identify potential structure-property relationships and elucidate complex design rules. However, experimentalists must note that predictive ML models cannot be used to make causal claims and any insight provided by such models should be validated through experimentation. Conversely, causal ML algorithms are designed to discover causal relationships^74^. These methods can be beneficial for causal inference but are often more difficult to implement and are only valid in certain settings. Thus, researchers should ensure that they fully understand how to properly implement these methods before using them for causal discovery. Within polymer design for regenerative medicine, Kumar et al. demonstrated the utility of SHAP and causal ML algorithms for identifying structure-property relationships associated with genetic cargo delivery from polymer systems^73^. Application of interpretable models, interpretation methods, and causal ML should be extended to other applications for biomedical polymers to further advance domain knowledge within these systems.

### Structure generation

**Fig. 3:**
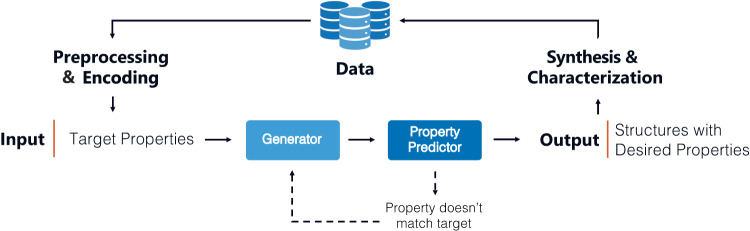
General ML workflow for inverse design approaches. Inverse design of polymers target algorithms which generate new, valid polymer structures with desired properties from property inputs. As seen in property prediction, data must be preprocessed & encoded prior to inverse design. Training involves the generation of a new structure through sequence perturbations or interpolations within existing latent spaces (represented here as ‘generator’). The properties of the new structure are predicted and compared to the target properties (shown as ‘property predictor’). The algorithms then iterate between these stages until structures with desired properties are achieved. While training of the ‘generator’ and ‘property predictor’ are approach-dependent, their hyperparameters may be tuned by minimizing the prediction error.

New polymer structures are often conceived from experimentalist intuition given their knowledge of existing work. Where property prediction tasks can be employed to screen candidates for accelerated materials design, the inverse design approach can train an algorithm which generates structures given desired properties (Fig. 3). This approach can overcome constraints introduced by the human imagination and preconceived beliefs about the complex structure-property relationships intrinsic to polymers. However, structure generation represents the most data hungry task due to the complexity of the models which can accomplish it; thus, big data availability presents a formidable obstacle.

The implementation of these inverse design approaches has been well discussed in other perspectives^75,76^. Briefly, the current efforts in the inverse design of polymer materials have followed a general workflow which iterates between generating new structures and predicting their properties (Fig. 3). This process proceeds by comparing the predicted properties to the target properties to minimize the difference between them. New structures can be generated through a sequence perturbation approach (e.g., Monte Carlo tree search, sequential Monte Carlo method, genetic algorithm) where a structure is incrementally changed to reach the property objective (“Generator” in Fig. 3)^33,77,78^. Alternatively, unsupervised methods (e.g., variational autoencoder) can be used to learn the structure-property latent space^25^. An interpolation method (e.g., linear interpolation) can then be used within regions which show higher probabilities for satisfying the property requirements to generate new structures. These candidate generation methods are then paired with other ML algorithms and/or molecular dynamics methods to predict their properties (“Property Predictor” in Fig. 3).

While success with these strategies has required considerable data (900–14,000 polymers), narrowing the scope of the inverse design problem to one class of polymers has proven successful on datasets as small as 171 polymers^25,33,78,79^. These current inverse design approaches have proceeded via similar workflows; thus, investigation into other inverse design algorithms (e.g., generative adversarial networks) represents a way to expand this field^80^. Ultimately, inverse design has the potential to accelerate the discovery of state-of-the-art materials, but is contingent on the creation of larger, more comprehensive datasets and/or the development of methods which can tolerate smaller, potentially imbalanced datasets.

### ML for polymer chemistry in medical applications

#### Current work

Mathematical modeling has a well-established role in understanding the drug release profile from polymer systems; thus, it is no surprise that work at the intersection of ML and medically-relevant polymers has focused on drug release behaviors (Fig. 1)^81–85^. These approaches primarily target candidate screening methods through property prediction algorithms including perturbation theory machine learning (PTML), light gradient boosting machine (GBM), bagged multivariate adaptive regression splines (MARS), and random forests. Additionally, property prediction has been a dominant approach toward predicting surface adsorption/attachment behavior for antifouling coatings to aid in candidate screening or elucidating quantitative structure property relationships^86–88^.

ML has also been applied toward the 3D printing conditions of tissue engineering scaffolds, 3D printing conditions of carbon doped polylactic acid for implantable biosensors and favorable conditions to generate medically-relevant microparticles^89,90^. Each of these approaches is underpinned by a focus on process optimization applied to a very narrow class of polymers with respect to structural diversity. Narrowing the scope of the input data accommodates a smaller number of observations which is compatible with the reality that biomedical properties are not as widely characterized as thermomechanical properties. Process optimization approaches also use less complicated inputs, such as concentration and printing speed, which facilitates the generation of larger datasets, more quickly when compared to chemical structure inputs which require synthesis to generate new observations.

**Fig. 4:**
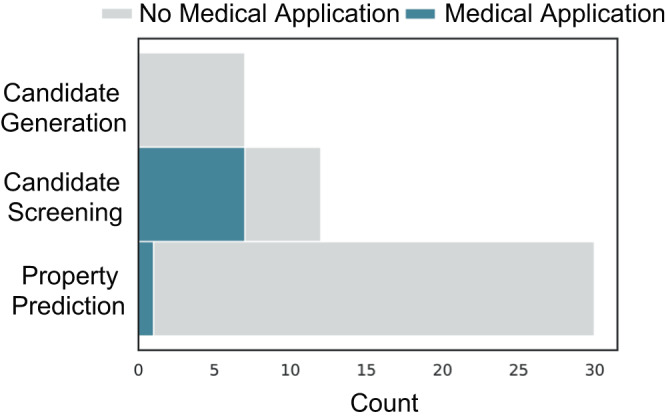
Uses for ML applied to polymer design. In each case, biomedical polymers represent a minority of the approaches and are notably missing from candidate generation strategies. The candidate screening methods shown here use property prediction algorithms to choose promising polymers out of a subset of interest. Thus, they differ from the property prediction category only in that their implementation goes a step further.

Examples of ML applied to the synthesis of new biomedical polymers are scarce (Fig. 4). Efforts in high-throughput, combinatorial design of copolymers have led to discoveries in polymer-mediated ribonucleoprotein delivery, antibiofouling hydrogels, polymer-protein hybrids, and ^19^F MRI agents^36,91–93^. These approaches leverage a limited compositional space and fast, simultaneous experiments to overcome data availability concerns. Additionally, as demonstrated by Reis et al., active learning can improve model performance considerably with only a few additional observations^36^. The compatibility of active learning with current experimental workflows makes it a promising way to introduce machine learning into existing polymer design problems.

#### Areas of need

The prediction of polymer properties that are unique to biomedical applications has lagged behind other achievements in ML property prediction (Fig. 4). Designing novel biocompatible polymers would benefit greatly from the ability to accurately predict cytotoxicity and bioactivity as demonstrated by previous work predicting nanoparticle toxicity^94,95^. The long timescale of data collection and non-standardized experimental methods to determine biocompatibility limit the generation of ML-viable datasets (Fig. 1 shows the relatively small size of biomedical data sets compared to non-biomedical applications).

Additionally, understanding the relationship between polymer degradation rate and underlying chemical structure is essential to tailoring biomedical polymers to their functionality and lifetime in the body. Physiologically degradable polymer systems improve patient quality of life by eliminating the need for secondary surgeries and reducing the possibility of long-term complications, such as infection at the implant site. However, there are often differing degradation behaviors in vivo vs. in vitro which adds to the necessary time and resources required to develop new physiologically degradable polymers^96,97^. Inverse design approaches will greatly expediate the process of finding polymer structures with the desired biocompatibility and degradation timescale. Investigation into other ML methods which accommodate smaller datasets such as active learning, physics-informed and interpretable models represent current steps that can be taken to augment existing design approaches. Interpretable models, particularly, are promising for advancing our fundamental knowledge of the structure-property relationships which govern these systems in addition to augmenting experimental efficiency.

There is significant potential for the ML methods described herein to accelerate discoveries for biocompatible polymers in a variety of biomedical research areas.

##### Drug delivery and polymer excipients

Extensive research has focused on developing drug release systems that pair highly tunable polymeric carriers (e.g. hydrogels, films, fibers), with target small molecules^98–100^. The interaction of the carrier with the desired drug greatly impacts drug loading, long-term shelf stability, and ultimately release profile^101^. Polymers are also employed as excipients in drug formulations which require materials that can reduce drug-drug interactions^42,102^. Whereas current work has focused on adjusting parameters of existing polymer systems, little emphasis has been placed on ML polymer design with precise drug release profiles, degradation behavior, and stimuli-responsiveness. Drug delivery and polymer excipients represent a promising area for ML to flourish in polymer design due to the small polymer quantity requirements associated with characterizing the properties of interest. In the short-term, existing high-throughput methods for the design of polymer excipients by Mann et al. could be extended to generate a dataset large enough for ML to be applied meaningfully^42^.

##### Regenerative medicine scaffolds

ML has been employed in the process optimization of scaffold preparation (e.g., bioprinting, electrospun fibers), but remains largely unexplored with regards to compositional landscape. Notably, there is a lack of biocompatible conducting materials for use in tissue scaffolds that mimic native tissue electrical properties^67^. It is also particularly challenging to quantify, and thus learn/predict some of the critical properties of polymeric scaffolds, including cellular proliferation and differentiation^103,104^. In particular, properties of interest to scaffolds for ML prediction will include cellular adhesion and in-growth, porosity, and tissue mechanics^105^. While data availability limits what can currently be done in polymer design for scaffolds, mechanical properties are more widely characterized and are relevant for this application. Thus, existing datasets which contain a broader scope of polymers could be adapted to ML approaches for polymer scaffold design.

##### Biologic sensing

Polymers have been used in biomedical sensing applications as both polymer electronics and encapsulations of traditional electronic sensors^106–108^. As the former, polymeric systems have been developed for strain and pressure sensing in applications such as cardiac monitoring and intracranial pressure. Although deep learning algorithms (e.g., CNN, HMM) have been applied to sense outputs such as electrocardiogram data, there have not been systematic studies relating material composition and device shape to targets, such as sensitivity to external stimuli and conductivity^109^. As encapsulations, properties such as inertness, water absorption, and water barrier performance will greatly benefit from ML prediction and, ultimately, the inverse design of novel polymer systems with these properties. More widely characterized properties like conductivity and mechanical properties are also relevant for sensing applications. Thus, as with biomedical scaffolds, existing datasets may be adaptable for this application.

##### Challenges and future directions

Current datasets for medically relevant parameters, such as degradation time and water uptake, are small, often exhibit non-standardized methods of characterization and have not been publicly assembled. Thus, the extension of ML methods to designing medically relevant polymers will require a paradigm shift in data curation. More intentional data curation by journals or data sharing by researchers can expand the possibilities of ML with medically relevant polymer synthesis without having to extract data by web scraping, which remains an ethically ambiguous task^110–112^. CRIPT is one promising data sharing platform to this end pending researchers’ engagement. The development of molecular dynamic simulation methods and high-throughput synthesis and characterization would make applying ML to biomedical polymers possible within individual groups. Standardization with respect to characterization, data analysis and data presentation of biomedically important properties, such as biocompatibility and degradation time, would also make accumulating larger datasets more feasible.

In addition to data availability concerns, more experimental validation of ML tasks would help establish the utility of these approaches in a wet-lab environment as well as provide insight into the performance of the model. Current work in polymer chemistry more broadly has focused on widely available properties (e.g., thermomechanical properties) and properties which are easily simulated (e.g., bandgap)—some of which are relevant to biomedical applications.

Extending this existing work to new fields is an achievable way to make progress toward expediting polymeric biomaterial design. Ultimately, applied ML represents an extremely promising tool toward accessing state-of-the-art of medically relevant polymers. This reality can be accelerated through:

I. Widespread contribution by experimentalists to data sharing platforms like CRIPT.
II. Standardization of the characterization of biomedically relevant properties (e.g., degradation, water uptake).
III. Development of affordable, high-throughput methods for polymerization via step-growth mechanisms (i.e., the majority of degradable polymers and minority of high-throughput approaches).
IV. Incorporation of coding proficiency and an introduction to ML methods as a part of chemistry curriculum.
V. Collaboration with ML experts and integration of ML methods into current research efforts.
